# BTK Inhibitors for the Treatment of Mantle Cell Lymphoma—Current Status and Perspectives

**DOI:** 10.3390/cancers18162698

**Published:** 2026-08-20

**Authors:** Tadeusz Robak, Anna Wolska-Washer, Paweł Robak

**Affiliations:** 1 Department of Hematology, Medical University of Lodz, 93-510 Lodz, Poland; adablju@tlen.pl (A.W.-W.); pawel.robak@umed.lodz.pl (P.R.); 2 Department of General Hematology Internal Medicine, Copernicus Memorial Hospital, 93-510 Lodz, Poland; 3 Department of Hematooncology and Internal Medicine, Copernicus Memorial Hospital, 93-510 Lodz, Poland

**Keywords:** BTK, acalabrutinib, degraders, ibrutinib, inhibitors, mantle cell lymphoma, pirtobrutinib, zanubrutinib

## Abstract

The introduction of Bruton’s tyrosine kinase inhibitors (BTKis) has significantly improved prognosis in the treatment of MCL. BTK inhibitors have demonstrated strong activity in the treatment of relapsed/refractory patients with mantle cell lymphoma (MCL), and several trials indicate that they also have potential as first-line treatment. Furthermore, combining BTKis with immunochemotherapy has enabled time-limited therapy as an alternative for continuous treatment with BTK inhibitors alone. In 2013, ibrutinib became the first BTK inhibitor to be approved by the FDA for previously treated MCL. The TRIANGLE study found ibrutinib to improve the efficacy of standard immunochemotherapy and reduce the need for autologous stem cell transplantation (ASCT) in younger patients; however, its findings do not conclusively confirm whether ASCT enhanced the activity of the ibrutinib-containing regimen in treatment-naïve patients. The second-generation covalent, irreversible BTK inhibitors acalabrutinib and zanubrutinib demonstrate greater selectivity and better pharmacological characteristics than ibrutinib. The FDA has approved acalabrutinib and zanubrutinib as single drugs for the treatment of R/R patients with MCL who have received at least one prior therapy. Acalabrutinib combined with bendamustine and rituximab was also approved for TN MCL unsuitable for ASCT. Pirtobrutinib, a first-in-class noncovalent reversible BTK inhibitor, was approved for the treatment of R/R MCL patients, including those resistant to covalent BTK inhibitors. Several other covalent and noncovalent BTK inhibitors are currently under investigation in MCL. Finally, BTK degraders have entered early clinical trials in B-cell lymphoid malignancies, and some of them in MCL.

## Simple Summary

The treatment of mantle cell lymphoma has been significantly improved by the introduction of Bruton’s tyrosine kinase inhibitors. Among the approved covalent inhibitors, acalabrutinib and zanubrutinib are more selective and better tolerated than ibrutinib; as such, they have become more widely adopted in treatment. The only noncovalent inhibitor approved for treating mantle cell lymphoma is pirtobrutinib, which is mainly used in patients with C481-mutated kinase. Currently, Bruton’s tyrosine kinase inhibitors are mainly used for the treatment of relapsed and refractory patients with mantle cell lymphoma who have received at least two prior therapies.

## 1. Introduction

Mantle cell lymphoma (MCL) is an aggressive non-Hodgkin lymphoma (NHL) characterized by the malignant transformation of B lymphocytes. MCL is characterized by heterogeneous clinical behavior, variable morphological characteristics. and generally poor prognosis [1]. The condition accounts for approximately 6% of all NHLs [2]. A diagnosis is confirmed by the presence of translocation t (11;14) (q13;q32), overexpression of cyclin D1, and a characteristic immunophenotype with expression of CD5, CD19, and CD20 and lack of expression of CD23, CD10, and BCL6 [3,4].

The prognosis of MCL is variable, with median overall survival ranging from 1.8 to 9.4 years, depending on various laboratory and clinical factors. The most important factors in stratifying patients into risk categories and choosing therapeutic options are the International Prognostic Index (MIPI) and Ki-67 [2]. Approximately 20% of patients with MCL present a more indolent course known as indolent non-nodal leukemia [5]. A worse prognosis is indicated by the presence of blastoid or pleomorphic variants, a high Ki-67 expression (≥30%), and the presence of *TP53* mutation or deletions. The choice of treatment for MCL patients depends on the aggressiveness of the disease, staging, MIPI, patient age, and comorbidities [6,7,8].

Asymptomatic patients with a low burden of lymphadenopathy, with no significant splenomegaly or cytopenias, may benefit from a watchful waiting strategy until progression. In treatment-naïve (TN) younger patients, immunochemotherapy based on high-dose cytarabine followed by autologous stem cell transplantation (ASCT) is an acceptable treatment option. In older, unfit patients, the commonly recommended therapy is R-CHOP (rituximab, cyclophosphamide, doxorubicin, vincristine, and prednisone) or bendamustine and rituximab (BR) followed by maintenance with rituximab [8,9].

More recently, targeted drugs have been introduced for the treatment of TN and relapsed/refractory (R/R) patients with MCL. Bortezomib combined with rituximab, cyclophosphamide, doxorubicin, and prednisone (VR-CAP) has been approved for older TN patients; the results of the LYM-3002 trial indicate that VR-CAP significantly prolonged OS compared to R-CHOP, with an acceptable safety profile [10,11].

Another targeted drug investigated in MCL is lenalidomide [12,13,14,15]. In patients with R/R MCL ineligible for intensive chemotherapy or stem cell transplantation, lenalidomide monotherapy was found to have an acceptable safety profile and induce longer PFS compared with the investigator’s choice, i.e., either rituximab, gemcitabine, fludarabine, chlorambucil, or cytarabine [12,15]. The combination of lenalidomide with rituximab (LR), used as initial treatment or in R/R patients, induces a high overall response rate (ORR) and durable remissions [13,15]. Finally, lenalidomide combined with BR (LBR) is an active regimen in elderly patients with MCL [14]. However, these treatments are associated with an unfavorable safety profile, including a high infection rate and second primary malignancies.

The introduction of Bruton’s tyrosine kinase inhibitors (BTKis) to the treatment of MCL has significantly improved the prognosis (Table 1) [16]. These drugs are divided into covalent irreversible BTKis (cBTKis), such as ibrutinib, acalabrutinib, and zanubrutinib; and noncovalent, reversible BTK inhibitors (ncBTKis), such as pirtobrutinib and nemtabrutinib [17,18]. Covalent BTK inhibitors bind permanently to the C481 residue of the BTK active site. Noncovalent BTK inhibitors do not bind to the C481 residue, and they are effective in patients with BTK C481 mutation. Acalabrutinib and zanubrutinib are more selective and better tolerated than ibrutinib, and they are now more commonly used in the treatment of MCL [19]. Pirtobrutinib is the only ncBTKi approved for the treatment of MCL [20]. However, most patients treated with BTK inhibitors will relapse, and some may become refractory to BTK inhibitor therapy. Resistance to cBTK inhibitors (ibrutinib, acalabrutinib, and zanubrutinib) in MCL may occur through a variety of mechanisms, including various genetic loci, such as BTK C481 mutations, as well as novel non-C481 mutations and *PLCG2* mutations. The C481 mutation results in substitution of the critical cysteine residue required for irreversible binding of cBTKis, thereby preventing effective inhibitor binding. In addition, gain-of-function mutations in PLCG2 activate B-cell receptor (BCR) signaling independently of BTK, allowing downstream signaling to bypass BTK inhibition and thereby confer resistance to BTKis. In these patients, the noncovalent BTK inhibitor pirtobrutinib is a suitable therapeutic option. Another potential therapeutic approach for patients with MCL who have developed resistance to BTKis could be the use of BTK degraders. BTK protein degraders are targeted drugs that exert their therapeutic effects by triggering ubiquitination of BTK, which targets the protein for degradation by the ubiquitin–proteasome system, thereby eliminating BTK from the cell [21,22]. BTK degraders have demonstrated adequate oral bioavailability and sustained pharmacodynamic target suppression in practice, confirming that the pharmacological barriers appear manageable [23]. Tacabrutideg (BGB-16673), NX-2127, and bexobrutideg (NX-5948) have shown promising results in Phase 1/2 clinical trials for R/R B-cell malignancies.

However, patients with BTKi-resistant MCL constitute a high-risk clinical group and urgently require effective therapy.

The aim of this review is to present the current status and future directions of BTK inhibitors in the treatment of MCL.

**Figure 1:**
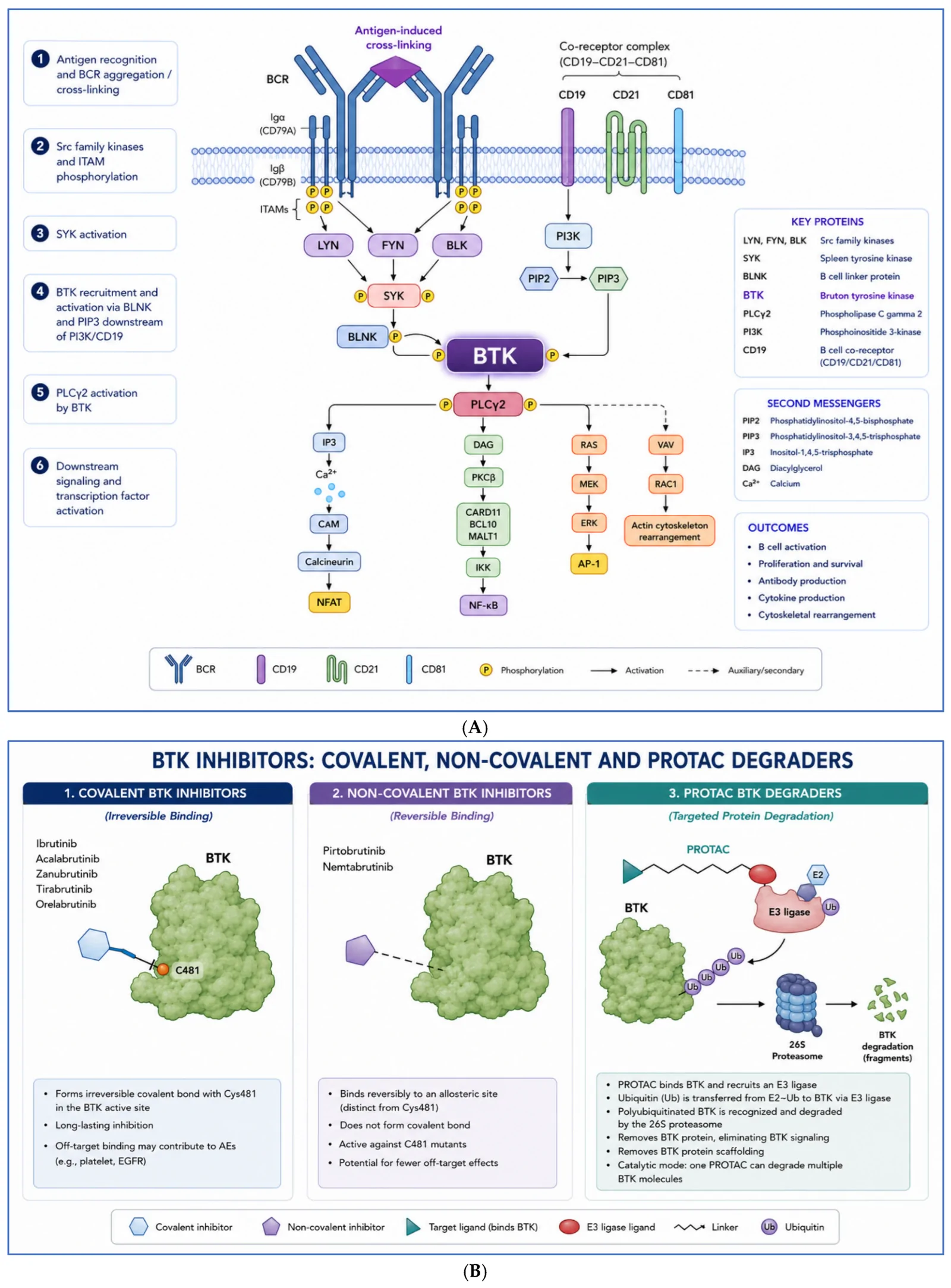
(**A**) BCR signaling. (**B**) BTK inhibition and degradation. Abbreviations: AP-1—activator protein 1, BCL—B-cell lymphoma, BCR—B-cell receptor, BTK—Bruton’s tyrosine kinase, CAM—multifunctional calmodulin kinase, CARD—caspase recruitment domain, DAG—diacylglycerol kinase, ERK—extracellular signal-regulated kinase, IKK—nuclear factor-κB kinase, ITAM—immunoreceptor tyrosine-based activation motif, LYN—non-receptor tyrosine-protein kinase, MALT—mitogen-activated protein kinase, MEK—mitogen-activated protein kinase, NFAT—nuclear factor of activated T cells, NF-κB—nuclear factor kappa-light-chain-enhancer of activated B cells, SYK—spleen tyrosine kinase, PI3K—phosphatidylinositol 3-kinase, RAC1—Ras-related C3 botulinum toxin substrate 1, RAS—rat sarcoma protein, VAV—guanine nucleotide exchange factor.

Figure 1 presents a schematic overview of BCR-driven BTK signaling and BTK-targeted therapeutic strategies, including cBTKi and ncBTKi as well as PROTAC-mediated BTK degradation.

## 2. Ibrutinib

Ibrutinib (Imbruvica, PCI-32765, Pharmacyclics, Bay Area, CA, USA and Johnson & Johnson, New Brunswick, NJ, USA) is the first-in-class cBTKi approved for the treatment of B-cell lymphoid malignancies, including MCL and chronic lymphocytic leukemia (CLL) (Table 1) [35,36]. In one Phase 2 registration trial, ibrutinib (560 mg) was evaluated in 111 MCL patients who had received one prior therapy (Table 2) [24]. The overall response rate (ORR) was 67% including 23% complete responses (CRs), with a median response duration 17.5 months. The 24-month progression-free survival (PFS) was 31%, and overall survival (OS) was 47%. The most common adverse events (AEs) were diarrhea, fatigue, nausea, and dyspnea. In another Phase 2 study performed in 16 R/R Japanese patients with at least one prior treatment regimen, ibrutinib (560 mg) was given once daily until relapse, progression, or unacceptable toxicity [37]. The ORR was 87.5%, and CR was 12.5%; however, 50% of the patents had at least one grade 3 AE, and 31.3% patients had serious adverse events (AEs), most commonly diarrhea and stomatitis, platelet count decrease, and anemia.

The combination of ibrutinib with rituximab was evaluated in 50 patients with R/R MCL at the MD Anderson Cancer Center as part of a phase 2 study (Table 2) [38]. Ibrutinib was given at a dose of 560 mg/day until disease progression or unacceptable toxicity. Rituximab was administered at 375 mg/m^2^/week for four weeks in cycle 1 and one dose on day 1 of each week for six cycles (i.e., cycles 3 to 8). Subsequently, rituximab was given at one dose every two months for up to two years [38]. At a median follow-up of 16.5 months, the ORR was 88%, with 44% CR. Some adverse effects were noted: grade 3 atrial fibrillation (AF) was observed in 12%, and grade 4 diarrhea and neutropenia were each observed in one patient. At a four-year follow-up, with a 16-month median treatment duration, 29 of 50 patients (58%) achieved CR, 38 patients had discontinued treatment, and 12 were still receiving treatment (Table 2) [39].

Even better results were observed in TN patients. Ibrutinib combined with rituximab without chemotherapy was evaluated in two small Phase 2 studies performed in TN MCL older patients, mainly at low risk (Table 2). In the first study, ORR was achieved in 84% of the patients, with a CR of 80% [51]. Undetectable MRD in peripheral blood was noted in 87%, with ibrutinib being discontinued in 69% of them. Treatment was generally well tolerated, and one patient developed severe aplastic anemia. Similar results were achieved in the second study, in older, previously untreated older patients (Table 2) [40]. Ibrutinib was administered with rituximab for two years, and then ibrutinib alone was continued until progression or unacceptable toxicity. The best ORR was 96%, including 71% CR. The three-year PFS was 87% and OS was 94%. The treatment was well tolerated, although grade 3 AF was observed in 11 (22%) patients.

A UK study evaluated ibrutinib, alone or in combination with rituximab, in TN patients. The ORR was 71.2%, the CR was 20.2%, and the median PFS was 26.0 months (Table 2) [41]. In the ENRICH study, ibrutinib combined with rituximab was compared with rituximab plus chemotherapy (RCHOP or BR) in 397 TN MCL patients aged 60 years and older; the ibrutinib–rituximab (IR) group achieved superior PFS to the immunochemotherapy group (Table 2) [42]. Also, at the median follow-up point (47.9 months), disease progression was observed in 94 (47%) of the 199 patients treated with ibrutinib and rituximab, and in 121 (61%) of 198 in the control group, with some deaths. The median PFS was 65.3 months in the ibrutinib plus rituximab group and 42.4 months in the RCHOP or BR group. The two groups achieved similar CR rates and similar 5-year OS (Table 2). However, the ibrutinib and immunochemotherapy arms were characterized by different grade 3 or higher AEs. Neutropenia was reported in 9%, of patients treated with the ibrutinib plus rituximab arm, 21% in the RCHOP arm, and 19% in the BR arm; grade 3 or higher hypertension was reported in 11% of IR, 4% of RCHOP, and 1% of BR. Atrial fibrillation was noted in 7% of patients in the IR group and 1% in the immunochemotherapy group. These results indicate that ibrutinib–rituximab should be considered as a treatment option in TN patients unsuitable for intensive treatment. Finally, ibrutinib combined with BR was compared with BR alone in TN MCL in a randomized Phase 3 study (Table 2) [43]. It was found that ibrutinib with BR prolonged PFS but not survival, and that combined treatment was more toxic than BR.

The efficacy and safety of ibrutinib monotherapy was compared with temsirolimus in a Phase 3 study including 280 patients with R/R MCL (Table 2) [44]. The ibrutinib arm demonstrated a longer median duration of OS (14.6 months) than the temsirolimus arm (6.2 months) and was better tolerated than temsirolimus. Grade 3 or higher treatment-emergent AEs were reported in 68% of patients in ibrutinib and 87% in temsirolimus, and treatment discontinuation due to AEs were noted in 6% and 26%, respectively.

Another Phase 2 study compared ibrutinib plus venetoclax with ibrutinib monotherapy in historical controls in 23 patients with R/R MCL and one TN patient (Table 2) [45]. At week 16, the combined therapy achieved a higher CR rate (42%) than the controls (9%; *p* < 0.001). For R/R MCL, the estimated 7-year PFS was 30%, and OS was 43%; the median PFS was 28 months [46]. Another Phase 1/2 trial evaluated ibrutinib, obinutuzumab, and venetoclax in R/R and TN patients with MCL (Table 2) [47]. The 2-year PFS was 69.5% and OS was 68.6% in R/R patients, while the 1-year PFS was 93.3% and OS was 100% in TN patients.

In the PHILEMON Phase 2 study, Jerkeman et al. evaluated the combination of ibrutinib, lenalidomide, and rituximab in 50 R/R MCL patients [48]. At a median follow-up of 17.8 months, the ORR was 76%, including 56% CR. The most common AEs were neutropenia, infections, and skin changes (Table 2) [48,49].

The three-arm, randomized, open-label, Phase 3 TRIANGLE study compared ibrutinib plus immunochemotherapy, with or without autologous ASCT, with immunochemotherapy and ASCT in TN patients with MCL (Table 2) [50]. It was found that combining ibrutinib with standard immunochemotherapy improves the efficacy of treatment and reduces the need for ASCT in MCL patients aged 65 years or younger. However, it remains uncertain whether ASCT enhanced the effect of the ibrutinib-containing regimen. A pooled analysis of clinical trials comprising 370 MCL patients treated with ibrutinib found the drug to be more effective when used earlier during treatment [52]. Similar results were observed in patients with CLL [53].

Ibrutinib was approved by the Food and Drug Administration (FDA) for previously treated MCL in 2013, and for R/R CLL in 2014 [54]. However, ibrutinib is frequently discontinued due to adverse events (AEs), including bleeding, cytopenias, and cardiac complications, particularly AF [24,55,56].

## 3. Acalabrutinib

Acalabrutinib (ACP-196, Acerta Pharma BV, Oss, The Netherlands; Calquence, AstraZeneca, Cambridge, UK) is a second-generation covalent, irreversible BTK inhibitor (Table 1). It has been found to demonstrate greater selectivity and better pharmacological characteristics than ibrutinib, with rapid oral absorption and a shorter plasma half-life. It is currently one of the most widely used drugs for the treatment of B-cell malignancies including CLL/SLL, MCL, Waldenstrom macroglobulinemia (WM), and marginal zone lymphoma (MZL) [57].

Acalabrutinib showed high activity and good tolerability as both a single drug and in combination with others in R/R and TN MCL (Table 3) [26,27,58,59,60,61,62,63]. When used as a single drug in R/R MCL, it showed high efficacy and an acceptable safety profile. In a Phase 2 trial (ACE-LY-004) comprising 124 R/R patients with a median age of 68 years and a median of two previous lines of treatment, acalabrutinib was administered at 100 mg twice daily (BID), until disease progression or unacceptable toxicity (Table 3) [58,59]. The ORR was 81%, with 40% CR. At a median 26-month follow-up, the median PFS was 20 months. Treatment was well tolerated. The most common all-grade AEs included headache, diarrhea, fatigue, and myalgia. Grade ≥ 3 AEs included neutropenia, anemia, and pneumonia. Bleeding was reported in one patient, and AF was not observed. Treatment discontinuation was reported due to progressive disease in 44% of cases, and due to AEs in 8%. In the final analysis, 43 (35%) patients had died: 29 (23%) due to progressive disease and six (5%) due to AEs. In October 2017, the FDA approved acalabrutinib as a single drug for the treatment of R/R patients with MCL who have received at least one prior therapy, based on the ACE-LY-004 trial [26]. Acalabrutinib-based combination therapies demonstrated strong efficacy in OS, CR, and PFS, with acceptable toxicity, supporting its use in TN patients. In January 2025, the FDA approved acalabrutinib combined with bendamustine and rituximab for TN MCL patients who are unsuitable for ASCT [27].

## 4. Zanubrutinib

Zanubrutinib (BGB-3111, Brukinsa^®^, BeOne Medicines, Cambridge, MA, USA) is another second-generation irreversible BTK inhibitor. In contrast to ibrutinib, it demonstrates more selective BTK binding and has been found to be active in B-cell lymphoid malignancies (Table 1). It is also less toxic than ibrutinib, demonstrating lower activity against EGFR, FGR, FRK, HER2, HER4, ITK, JAK3, LCK, BLK, and TEC kinases [64]. Zanubrutinib also exhibits higher bioavailability than ibrutinib and, unlike ibrutinib, enables therapeutic levels above the half-maximal inhibitory concentration (IC50), which can be maintained during daily and twice-daily administration [64,65]. Zanubrutinib has been compared head-to-head with ibrutinib in CLL and WM [66,67]. Both trials showed that zanubrutinib has better efficacy and tolerability, particularly a lower risk of AF and major bleeding events. However, there is no similar study directly comparing zanubrutinib with ibrutinib in MCL.

A Phase 1/2 study (NCT02343120) examined the effects of zanubrutinib at 160 mg twice daily and 320 mg once daily in patients with R/R MCL [28,68]. At a median follow-up of 18.8 months, the ORR was 84%, including 25% CR and median PFS of 21.1 months. The OS at 24 months was 64.4%. In addition, 86 patients with R/R MCL were treated with zanubrutinib at 160 mg twice daily as part of a multicenter, open-label, Phase 2 study [68]. At a median follow-up of 35.3 months, the ORR was 83.7%, including 77.9% CR, with median PFS of 33.0 months. The 36-month PFS was 47.6%, and the OS rate was 47.6%. The most common grade ≥ 3 AEs were neutropenia (18.6%) and pneumonia (12.8%). No cases of AF or grade ≥ 3 cardiac events were observed. The recommended dose of zanubrutinib in MCL is either 160 mg twice daily or 320 mg once daily.

A retrospective analysis by Philips et al. compared the efficacy of the cBTKis ibrutinib, zanubrutinib, and acalabrutinib, used as single drugs, in 698 R/R MCL patients in second- or third-line scenarios [36]. The median time to next treatment (TTNT) was 14.5 months for second-line zanubrutinib, 12.8 months for acalabrutinib, and 10.3 months for ibrutinib. The median PFS was 26.4, 23.2, and 29.3 months, respectively. Median OS was not reached for zanubrutinib, 27.4 months for acalabrutinib, and 27.0 months for ibrutinib. Currently, a regimen based on zanubrutinib plus rituximab followed by zanubrutinib monotherapy, followed by observation, is under comparison with BR in an ongoing Phase 3 study (NCT04002297). The cohort comprises untreated MCL patients unsuitable for autologous SCT [69]. However, caution should be exercised in the cross-trial comparisons, as the data are based on separate clinical trials with differing patient populations and study designs. Furthermore, there may also be other factors that are as-yet not understood that may affect the efficacy and toxicity results.

Finally, a Phase 2 study has evaluated zanubrutinib, obinutuzumab, and venetoclax (BOVen) in 52 TN patients with TP53-mutated MCL (NCT03824483) [70]. Zanubrutinib was given at 160 mg twice daily, and obinutuzumab at 1000 mg on days 1, 8, and 15 of cycle 1, and on day 1 of cycles 2 to 8. Venetoclax was added after two cycles, with the dose increasing weekly to 400 mg daily. Treatment was discontinued after 24 cycles, if undetectable measurable residual disease (uMRD) was achieved in an immunosequencing assay. The results indicate ORR in 96% patients, including 88% CR (22/25). At cycle 13, uMRD was detected at a sensitivity level of 1 × 10^−5^ in 95% patients and of 1 × 10^−6^ in 84%. At a median follow-up of 28.2 months, the 2-year PFS was 72%, and OS was 91%. Elsewhere, a Phase 1 study investigated the combination of zanubrutinib with zandelisib, a phosphatidylinositol 3-kinase delta (PI3Kδ) inhibitor, in previously treated follicular lymphoma (*n* = 31) or MCL (*n* = 19) [71]. Treatment was associated with high response rates, and no significant toxicity was noted for either agent. The OR rate was 87% (CR = 33%) for FL and 74% (CR = 47%) for MCL. At a median follow-up of 16.5 months for FL and 10.9 months for MCL, the estimated 1-year PFS was 72.3% and 56.3%, respectively.

## 5. Orelabrutinib

Orelabrutinib (ICP-022, HIBRUKA Biogen, Cambridge, MA, USA/Innocare Pharma, Beijing, China), is a highly selective, irreversible cBTK inhibitor designed to increase selectivity and reduce off-target side effects (Table 1) [72]. In a Phase 2 study including 97 patients, regimen selection was performed for 100 mg of BID and 150 mg of QD. The 150 mg QD dose was selected for further evaluation: OR was 87.9%, including CR 27.4% and DOR of 73.7% at 12 months [73]. At 12 months, the PFS rate was 70.8% and the OS rate was 88.7%. Orelabrutinib was well tolerated, with the most common treatment-related AEs being thrombocytopenia, neutropenia, leukopenia, and gastrointestinal toxicity. In a longer Phase 1/2 study, orelabrutinib was evaluated in 106 patients [29]. Of these, 86 patients received 150 mg once daily and 20 received 100 mg twice daily. After a median follow-up duration of 23.8 months, the ORR was 81.1%, including CR in 27.4%. The median response duration was 22.9 months, and the median PFS was 22.0 months; the OS at 24 months was 74.3%. The most common AEs were thrombocytopenia (34.0%), upper respiratory tract infection (27.4%), and neutropenia (24.5%). Orelabrutinib plus BR is currently being compared with BR alone in patients with treatment-naïve MCL as part of an ongoing Phase 3 trial (NCT06363994). In December 2020, orelabrutinib was approved in China for the treatment of patients with MCL and CLL/SLL after at least one prior treatment, and in June 2021 it was granted Breakthrough Therapy Designation for the treatment of R/R MCL by the FDA. In February 2021, Chinese regulators approved it for first-line MCL therapy in combination with R-CHOP chemotherapy.

## 6. Pirtobrutinib

Pirtobrutinib (LOXO-305, Jaypirca, Eli Lilly, Indianapolis, IN, USA) is a first-in-class reversible ncBTK inhibitor (Table 1) [20,74]. As the drug does not need to bind to the C481 residue, it can overcome resistance to cBTKis caused by the BTK C481S mutation. It has high oral bioavailability and a long half-life of approximately 19 h; its low off-target activity also minimizes side effects [75].

As part of the Phase 1/2 BRUIN (NCT03740529) trial, pirtobrutinib was investigated against R/R lymphoid malignancies in 52 evaluable patients with MCL, previously treated with cBTK inhibitors [76]. The drug was given at doses ranging from 25 to 300 mg QD in 28-day cycles in Phase 1, and at the recommended dose of 200 mg QD in Phase 2. The ORR among patients was found to be 52%. A subsequent study comprising 90 R/R MCL patients who had received a median of three prior lines of therapy achieved an OR rate of 57.8%, including CR in 20% and median PFS of 7.4 months. Of these patients, 82.2% had discontinued prior cBTKi treatment because of disease progression.

Another study of 124 patients with non-blastoid MCL examined data based on patient-reported outcomes [77]. Of 52 patients previously treated with cBTK inhibitors, the ORR was 52%. In addition, over 70% of patients demonstrated improvement or remained stable through cycle 20, and the median time to worsening was not reached. At a median follow-up of 39.7 months, nine patients with MCL remained under observation.

A real-life observation performed as part of a retrospective multicenter analysis in an Italian population (42 R/R patients) evaluated the use of pirtobrutinib at the approved dose (200 mg once a day; QD) as part of a compassionate use program [78]. The OR rate was found to be 47.6%, with CR in 23.8%. After a median observation of seven months, the median PFS was 4.7 months and the median OS was 15.3 months. A similar European study evaluated 10 patients who had previously received three lines of systemic therapy [79]. The OR rate was 67%, and neither mean duration PFS nor OS was reached at a median follow-up of 8.6 months.

The effects of pirtobrutinib treatment on R/R but BTKi-naïve MCL patients are currently under investigation in the BRUIN MCL-321 Phase 3 study (NCT04662255). The participants have been randomized to pirtobrutinib or the investigator’s choice of covalent BTKi. The results are not yet available. In January 2023, pirtobrutinib was approved by the US FDA for the treatment of R/R MCL patients, including those resistant to cBTK inhibitors [74].

## 7. BTK Inhibitors Investigated in MCL

Several unapproved cBTK and ncBTK inhibitors are currently under investigation in B-cell lymphoid malignancies to improve the safety and efficacy of approved drugs such as ibrutinib.

### 7.1. Tirabrutinib

Tirabrutinib (Velexbru^®^, ONO/GS-4059, Ono Pharmaceutical, Osaka, Japan/Gilead Sciences, Foster City, CA, USA) targets BTK C481 more specifically and selectively than ibrutinib and has demonstrated potent activity in patients with CLL/SLL (Table 1) [30]. The drug has been evaluated in an initial Phase 1 study (#NCT01659255) involving 90 R/R patients with various B-cell lymphomas, including 16 patients with MCL [80]. Responses were observed in five (31%) patients with MCL, with an estimated mean PFS of 874 days. In a longer follow-up, with a median treatment duration of 97.3 weeks, the ORR was found to be 68.8%, with 55% CR. Of the 16 MCL patients, 11 discontinued treatment, including nine for disease progression.

One meta-analysis examined tirabrutinib data from seven studies including patients with CLL, primary central nervous system lymphoma (PCNSL), MCL, and WM [81]. The pooled ORR was 72.5%, and the CR rate was 18.6%. Monotherapy was found to achieve a manageable safety profile and promising efficacy in patients with B-cell lymphoma. The most common all-grade AEs in patients with MCL were thrombocytopenia (44.0%), diarrhea (44.0%), and coughing (44.0%); the most common grade ≥ 3 AE was thrombocytopenia (19.0%). Patients with MCL achieved the highest CR (37.5%). There are currently no registered studies of tirabrutinib in MCL.

### 7.2. DTRMWXHS-12

The pyrazolo-pyrimidine derivative DTRMWXHS-12 (DTRM-12) was investigated as part of a Phase 1–2 study in patients with lymphoid malignancies (CLL and NHL), including three (9%) with MCL (Table 1). In the first-in-human Phase 1 part of the study, DTRMWXHS-12 was investigated in patients with R/R CLL and lymphomas as three regimens: monotherapy, in combination with everolimus, and with everolimus plus pomalidomide [31]. DTRM-12 monotherapy was well tolerated in B-cell malignancies and CLL, and no dose-limiting toxicity was observed. Pharmacokinetic (PK) studies demonstrated adequate target drug exposures at all doses (50 mg, 100 mg, 200 mg, and 300 mg). The ORR for all patients and doses was 13/31 (41.9%), including two patients (25%) treated with DTRM-12 only.

DTRMWXHS-12 is also under evaluation as part of an ongoing Phase 1b study in patients with R/R MCL (NCT03836768). The primary objective of the study is to evaluate the safety and tolerability of DTRMWXHS-12 and recommend the dosing method to be used in a later Phase 2 study. A single-arm, multicenter, open-label Phase 2 study was initiated in 2022 in China (NCT03836768, ChiCTR2200058983), with the aim being to evaluate the efficacy and safety of DTRMWXHS at doses of 150 mg and 300 mg in R/R MCL.

### 7.3. Nemtabrutinib

Nemtabrutinib (MK-1026, ARQ-531, Merck, Rahway, NJ, USA), like the ncBTKi pirtobrutinib, reversibly inhibits both wild-type and C481S-mutated BTK (Table 1). The drug was investigated in a Phase 1/2 dose escalation study encompassing 112 patients with R/R hematological malignancies (NCT03162536, ARQ 531-101/MK-1026-001) [32]. The most common treatment-related AEs were dysgeusia (21%) and neutropenia (20%). A Phase 2 study evaluating the safety and efficacy of nemtabrutinib in participants with hematological malignancies, including CLL/SLL and MCL, is ongoing, and the results are expected in 2029 (NCT04728893). The drug is also currently under evaluation as combined therapy in two Phase 2 studies. In the first, the Waveline-006 study (NCT05458297), it achieved an OR of 64% in combination with zilovertamab vedotin in patients with R/R MCL [33]. In the second (NCT06572618), in combination with rituximab in patients with TN MCL, the results are still anticipated.

### 7.4. Rocbrutinib

Rocbrutinib (LP-168, HS-10561; NWP-775; Hansoh Pharma, Lianyungang, China) is a highly selective fourth-generation dual BTK inhibitor demonstrating both covalent (irreversible) and noncovalent (reversible) binding (Table 1) [82]. The ROCK-1 Phase 2 trial evaluated its safety and efficacy in 61 heavily pretreated patients with R/R MCL. The patients had previously been treated with cBTK inhibitors and received a median of three previous therapies (NCT05716087, LP-168-CN201). The results indicate ORR in 63.9% of patients, including CR in 23.0%, median PFS of 7.39 months, and 12-month estimated duration of response (DOR) of 61.2%. The most common treatment emergent AEs (TEAEs) were thrombocytopenia (43.5%), anemia (30.6%), neutropenia (29.0%), increased blood creatinine (21.0%) and hyperuricemia (21.0%), mainly at grade 1 or 2. Among special-interest AEs related to BTKis, major bleeding was noted in 3.2% of cases; no AF was observed. Rocbrutinib is currently under evaluation versus the investigator’s choice of BTK inhibitor (ibrutinib, acalabrutinib, zanubrutinib, or orelabrutinib) in R/R MCL as part of a Phase 3 study. Moreover, two Phase 2 studies of rocbrutinib in patients with R/R MCL are ongoing in China (NCT05716087, NCT07377578).

### 7.5. Vecabrutinib

Vecabrutinib (SNS-062, Sunesis Pharmaceuticals, San Francisco, CA, USA) is a selective, reversible, noncovalent BTK inhibitor that demonstrates antitumor activity, irrespective of Cys481Ser mutation [83]. The drug is being investigated in a Phase 1b/2 study in patients with various B-cell malignancies, including CLL (NCT03037645). In a Phase 1b dose-escalation study, vecabrutinib was well tolerated up to 410 mg twice daily (BID) [84]. However, clinical benefit was limited, and clinical trials were discontinued due to lack of efficacy.

## 8. BTK Degraders

Targeted protein degradation represents a promising therapeutic strategy that may overcome the limitations associated with BTKis. Proteolysis-targeting chimeras (PROTACs) are heterobifunctional molecules that induce selective degradation of target proteins by harnessing the ubiquitin–proteasome system, thereby enabling catalytic, event-driven protein degradation [85,86]. Unlike BTK inhibitors, PROTACs do not rely solely on binding to the BTK active site and, therefore, retain activity in patients harboring BTK mutations that confer resistance to BTKis [34,83]. BTK degraders are orally bioavailable small molecules that promote degradation of the BTK protein through the ubiquitin–proteasome system, resulting in sustained suppression of BTK signaling [21,87]. Three BTK degraders are under investigation in B-cell malignancies, *viz.*, tacabrutideg (BGB-16673, BeOne Medicines, Cambridge, MA, USA), bexobrutideg (NX-5948, Nurix Therapeutics, Brisbane, CA, USA), and NX- 2127 (Nurix Therapeutics, Brisbane, CA, USA) [88,89]. Tacabrutideg is under study in R/R CLL/SLL, WM, FL, and MZL in a Phase 1 study, and in CLL in a Phase 3 study [90].

Preliminary safety and efficacy data for tacabrutideg were recently presented following the Chinese Phase 1/2 CaDAnCe-102 study; the participants comprised patients with R/R B-cell malignancies, including 15 with MCL [25]. Three MCL patients had previously been treated with BTK inhibitors and achieved a CR, including one patient who was heavily pretreated, with seven prior lines of therapy. The most common grade ≥ 3 TEAEs were neutropenia and pneumonia. However, AF was not observed. These data support further studies of tacabrutideg in patients with MCL. The early reports indicate that BTK degraders have significant clinical activity in heavily pretreated patients with lymphoid malignancies. These agents will be probably used in double-refractory patients who have received cBTKis and nc BTKis.

## 9. Conclusions

Front-line chemoimmunotherapy and ASCT are well established in the management of younger, fit patients with MCL. However, in older, unfit patients, current treatment options are based around combinations of less-intensive chemotherapy and maintenance rituximab therapy. Patients who are not eligible for immunochemotherapy may also be suitable for lenalidomide and rituximab. The treatment of R/R MCL has been considerably improved by the development of covalent BTKis. Among them, ibrutinib, and the more specific second-generation BTKis acalabrutinib and zanubrutinib, have changed the landscape in the treatment of R/R MCL and are now recommended for MCL patients at first relapse after chemoimmunotherapy.

While new therapeutic options including BTK degraders and BCL-2 inhibitors are under evaluation, mature results from clinical trials remain unavailable. In early clinical trials, some BTK degraders have shown significant activity and acceptable tolerability in heavily pretreated MCL patients. Among them, tacabrutideg, bexobrutideg, and NX-2127 are the most advanced in trials and should be available for the treatment of MCL in the near future. Of the BCL2 inhibitors, possibly the most widely used is venetoclax, which has proven effective against MCL as a single agent and in combination.

The ongoing development of novel immunotherapeutic options, such as CAR T cells and T-cell-activating bispecific antibodies, also offers hope for MCL patients. Anti-CD19 chimeric antigen receptor (CAR) T-cell therapy is now included in the treatment regimens of several lymphoma subtypes, including MCL, and has been found to offer effective and durable clinical responses for BTKi-refractory MCL.

The selection of appropriate treatment for MCL relies heavily on prognostic factors such as the MCL-International Prognostic Index (MIPI), proliferation index Ki-67, and presence of TP53 aberrations. However, these are being supplemented by novel molecular and cytogenetic biomarkers for the selection of chemotherapy and novel agents, which may have important parts to play in making treatment decisions and identifying research directions.

## Figures and Tables

**Table 1:** Characteristics of BTK inhibitors approved and in clinical trials in MCL.

BTK Inhibitor	Characteristics	Selected Clinical Trials in MCL	FDA Approval for MCL	References
Ibrutinib (PCYC-1102, Imbruvica^®^, Johnson & Johnson)	First generation cBTKi	Phase 2 Trial PCYC-1104-CA: Ibrutinib has high efficacy and favorable safety profile in R/R MCL (NCT01236391). Phase 3 TRIANGLE Study: Adding ibrutinib to standard immunochemotherapy improves outcomes in younger patients (NCT02858258).	2013: approved after at least one prior therapy.	[24,25]
Acalabrutinib (ACP-196, Calquence^®^, AstraZeneca)	Second-generation cBTKi is more selective and less toxic than ibrutinib	Phase 2 ACE-LY-004, (NCT02213926) Trial: Acalabrutinib induces high rate of durable responses and a favorable safety profile in R/R MCL patients. Phase 3 ECHO Trial (NCT02972840): ABR vs. BR—longer PFS for ABR.	2017: approved in R/R MCL after at least one prior therapy; 2025: approved in combination with BR for TN MCL ineligible for ASCT.	[26,27]
Zanubrutinib (BGB3111, Brukinsa^®^, BeOne Medicines)	Second-generation cBTKi with greater specificity and better bioavailability compared with ibrutinib	Phase 1/2 Trial BGB-3111-AU-003 (NCT02343120): Zanubrutinib at 160 mg BID or 320 mg once daily in R/R MCL—ORR 84%, CR 25%, and median PFS 21.1 m.	2019: approved in monotherapy for R/R MCL after at least one prior therapy.	[28]
Orelabrutinib (ICP-022, HIBRUKA Biogen/Innocare Pharma)	Highly selective, cBTKi. Greater specificity and better bioavailability compared with ibrutinib	Phase 2 Study: Orelabrutinib in R/R MCL—ORR 87.9%, 12-month PFS 70.8% and OS 88.7%, no Gr 3 or higher diarrhea, AF, or severe bleeding.	2021: FDA granted a breakthrough therapy designation for R/R MCL.	[29]
Pirtobrutinib (LOXO-305, Jaypirca, Eli Lilly)	Highly selective, ncBTKi, inhibiting diverse BTK C481 mutations	Phase 1/2 BRUIN (NCT03740529) Trial: In patients with R/R MCL pretreated with cBTKi—ORR 57.8%, CR 20%, median PFS 7.4 m.	2023: FDA granted accelerated approval for R/R MCL after at least two lines of therapy, including cBTKi.	[20]
Tirabrutinib (Velexbru^®^, ONO/GS-4059, Ono Pharmaceutical, Gilead Sciences)	Second-generation, highly selective cBTKi with the ability to cross the blood–brain barrier	Phase 1 Study (NCT01659255): In R/R MCL—ORR 68.8%, CR 55%.	Not approved.	[30]
DTRMWXHS-12 (DTRM-12)	Pyrazolo-pyrimidine derivative cBTK inhibitor	Phase 1b Study MCL Initiated in 2022 in China: Doses of 150 mg and 300 mg in R/R MCL (NCT03836768, ChiCTR2200058983).	Not approved.	[31]
Nemtabrutinib (MK-1026, ARQ-531, Merck)	ncBTKi of both the wild-type and the mutation C481S of BTK	Phase 2 Waveline-006 Study (NCT05458297): Nemtabrutinib + zilovertamab vedotin in patients with R/R MCL—ORR—64%. Phase 2 Study (NCT06572618): Nemtabrutinib + rituximab in TN MCL	Not approved.	[32,33]
Rocbrutinib (LP-168, HS-10561; NWP-775; Hansoh Pharma	Highly selective 4th-generation dual BTKi with both cBTKi and ncBTKi.	Phase 2 Trial (ROCK-1, NCT05716087: Rocbrutinib in R/R patents with MCL—ORR 63.9%, CR 23.0%, PFS 7.39 months	Not approved.	[34]

Abbreviations: ABR—acalabrutinib + BR, AEs—adverse events, BID—twice daily, BR—bendamustine plus rituximab, BTKi—Bruton’s tyrosine kinase inhibitor, cBTKi—covalent BTKi, CR—complete response, Gr—grade, m—months, MCL—mantle cell lymphoma, ncBTKI—noncovalent BTKi, ORR—overall response rate, PFS—progression-free survival, R/R—relapsed/refractory, TN—treatment-naïve.

**Table 2:** A summary of key clinical trials evaluating ibrutinib as a single agent and in combination in MCL.

Study [Reference]	Patient Characteristics	Treatment	Median FU	Efficacy	Safety	Comments
Wang et al. 2015, Phase 2 [24]	*n* = 111, R/R	I 560 mg once daily, until progression or unacceptable toxicity	26 m	ORR 67%, CR 23%, 24 m PFS 31%, 24 m OS 47%	AEs: diarrhea (54%), fatigue (50%), nausea (33%), and dyspnea 32%).	Ibrutinib induces durable responses and favorable safety in R/R MCL.
Wang et al. 2016, Jain et al. 2018 Phase 2 [38,39]	*n* = 50, R/R	I + R	47 m	ORR 88%, CR 58%, median PFS 43 m	Gr 1–2 toxicities: fatigue, diarrhea, nausea, arthralgias, and myalgias. Gr 3 AF 12%.	Ibrutinib + rituximab is active and well tolerated in R/R MCL
Jain et al. 2022, Phase 2 [40]	*n* = 50, TN. Older, non-blastoid	I + R	45 m	ORR 96, CR 71%, 3-year PFS 87%, and OS 94%	Gr 3–4 AF 18%. Fatigue 14%, diarrhea 14%, anemia 8%, neutropenia 4%, thrombocytopenia 4%.	Ibrutinib + rituximab is an effective, easily administered, and safe option in elderly patients with non-blastoid MCL.
Tivey et al., 2024, real-word study [41]	*n* = 149, TN,	I +/− R (IR—39.0%)	15 m	ORR 71.2%, CR 20.2%, median PFS 26.0 m	Gr ≥ 3 all-cause toxicity 20.3%, Gr ≥ 3 bleeding 4.0%, Gr ≥ 3 non-neutropenic infection 7.4%, AF 6.6%	IR is effective and well tolerated in TN MCL; PFS and OS were inferior in high-risk disease
Lewis et al. 2025, phase 2/3 ENRICH study [42]	*n* = 397, phase 2/3, TN ≥ 60 yrs	I + R vs. immunochemotherapy (RCHOP 27% or BR 73%)	47 m vs. 9 m	ORR 86% vs. 85%, CR 54 vs. 53%; 5-year PFS: IR—52% vs. RCHOP 19%, IR 51% vs. BR 47%	Total AEs during induction: IR—42%, RCHOP 67%, BR 51%. All cardiac events: IR—11%, RCHOP 10%, BR 5%. All bleeding events: IR—3%, RCHOP 6%, BR 1%.	Ibrutinib + rituximab is suitable treatment for TN, older patients with MCL.
Wang et al. 2022, phase 3 SHINE tral, [43]	*n* = 523 TN, ≥65 yrs	I + BR vs. BR	84 vs. 7 m	Median PFS: 80.6 m vs. 52.9 m (*p* = 0.01), ORR: 89.7% vs. 88.5%; CR 65.5% vs. 57.6%,(*p* = 0.06)	Gr 3 or 4 AEs 81.5% vs. 77.3%.	Ibrutinib combined with BR significantly prolonged PFS.
Dreyling et al. 2016, phase 3 [44]	*n* = 280; R/R,	I vs. Memsirolimus	20 m	ORR: 72% vs. 40%, median PFS: 14.6 m vs. 6.2 m	Most common AEs: Ibrutinib: diarrhea 29%, cough 22%, fatigue 22%. Temsirolimus: thrombocytopenia, 56%, anemia 43%, diarrhea 31%, neutropenia 26%.	Ibrutinib treatment showed improvement in PFS and better tolerability versus temsirolimus in R/R MCL lymphoma.
Tam et al., 2018, phase 2 [45] Handunnetti et al. 2024 [46]	*n* = 24, R/R	I + Ven until progression	7 yrs	OR 71%, CR 62%, PFS at 15 m 78%, at 7-yrs 30%	Most common AEs: diarrhea 83%, fatigue 75%, nausea and vomiting 71%.	Ibrutinib plus venetoclax induces long-term durable responses and acceptable toxicity profile in R/R MCL.
Le Gouill et al. 2021, Phase 1/2 [47]	*n* = 48 R/R and TN	Cohort A: I + Ob R/R *N* = 9 Cohort B: I + Ob + Ven—R/R *N* = 24 Cohort C: I + Ob + Ven—TN *N* = 15	R/R 17 m, TN 14 m	R/R: ORR 84% CR 67%, 1-year PFS was 74.5%, OS 87.5% TN: ORR 100%, CR 86.6%; 1-yr PFS 93.3% OS 100%	Most frequent Gr 3/4 AEs in all cohorts: thrombocytopenia and neutropenia.	Obinutuzumab + ibrutinib + Ven induces high response rates with an acceptable safety profile.
Jerkeman et al. 2018 PHILEMON tral, phase 2 [48], Forsgren et al., 2025 [49]	*n* = 50 R/R phase 2	I + lenalidomide + R	92 m	ORR 76%, CR 56%; median PFS 17.4 m, median OS of 45.3 m	Most common Gr 3–4 AEs: neutropenia 38% infections 22%, cutaneous toxicity 14%.	Ibrutinib + lenalidomide + rituximab is active and well tolerated in R/R MCL.
Dreyling et al. 2024, phase 3 TRIANGLE [50]	*n* = 870 TN, <65 years	Group A: Chemo + ASCT Group B: I + ASCT Group C: I	31 m	3 Yr PFS: Group A: 72% Group B: 88% Group C: 86%	No relevant differences in Gr 3–5 AEs during induction or ASCT.	Adding ibrutinib during induction and maintenance should be part of treatment in younger TN pts.

Abbreviations: A acalabrutinib, AEs adverse events, AF—atrial fibrillation, ASCT—autologous stem cell transplantation, BID—twice daily, BR—bendamustine plus rituximab, BTKi—Bruton’s tyrosine kinase inhibitor, CR—complete response, Gr—grade, I—ibrutinib, IR—ibrutinib plus rituximab, m—months, M—month, MCL—mantle cell lymphoma, Ob—obinutuzumab, ORR—over all response rate, OS—overall survival, PFS—progression free survival, Pts—patients, R—rituximab, RCHOP—rituximab, cyclophosphamide, adriamycin, vincristine, prednisone, R/R—relapsed/refractory, TN—treatment-naïve, Ven—venetoclax, Yr—year.

**Table 3:** A summary of key clinical trials evaluating acalabrutinib as a single agent and combination in MCL.

Study [Reference]	Patient Characteristics	Treatment	Median FU	Efficacy	Safety	Comments
Wang et al. 2018 [26], Le Gouill et al. 2024 ACE-LY-004 [58] Phase 2,	*n* = 124, R/R	Acalabrutinib 100 mg × 2/d continuously	38.1 m	ORR 81.5%, CR 47.6%, median PFS 22.0 m, OS 59.2 m, 5-year OS 49.5%	AE of clinical interest: AF 2.4%; hypertension 4.0%, major hemorrhage 4.0%, infections 67.7%.	Study supports the use of acalabrutinib in patients with R/R MCL.
Wang et al. 2019 [59]	*n* = 50, TN, ≥65 yrs, phase 2	Acalabrutinib + rituximab	17 m	ORR 94%, CR 90%, 2 year PFS 92%, 2 year OS 96%	All-grade AEs: fatigue 82%, myalgia 64%, headache 38%, bruising 28%.	Acalabrutinib + rituximab is highly effective and safe treatment in older pts with MCL.
Phillips et al. 2025, (ACE-LY-106) [60] Phase 1b	*n* = 38 TN (*N* = 18) R/R (*N* = 20),	Acalabrutinib + BR	24 m	TN: ORR 94.4%, CR 77.8%, median PFS not reached R/R: ORR 85%, CR 70.0%, median PFS 28.6 m	Gr ≥ 3 AEs: TN 72.2% R/R 85.0%, most commonly neutropenia TN: 38.9%; R/R: 50.0%.	Acalabrutinib + BR demonstrates high efficacy in patients with TN and R/R MCL.
Wang et al. 2025, ECHO trial [27] phase 3	*n* = 598, TN, ≥65 years,	Acalabrutinib + BR vs. BR	49.8 m	ORR 91.0% vs. 88.0%; PFS 66.4 vs. 49.6 m, OS: HR 0.86 *p* = 0.27.	Gr ≥ 3A AEs: 88.9% vs. 88.2%, Gr ≥ 3 serious AEs 64.3% vs. 55.9%.	ABR improved PFS in older patients; OS was similar, but the majority of patients crossed over to treatment with a BTKi.
Wang et al. 2024, [61] Phase 1b	*n* = 21, TN	Acalabrutinib + venetoclax + rituximab	27.8 m	ORR 100% CR 71.4%, MRD 87.5%, PFS at 1 yr 90.5%, PFS at 2 year 63.2%	Any-grade AEs: diarrhea 71.4%, headache 52.4%, and fatigue 7.6%. Gr ≥ 3 AEs 61.9%, most commonly neutropenia (33.3%).	ART is a promising, highly effective, and well-tolerated chemotherapy-free treatment option for TN MCL.
Kim et al. 2025, MAVO, [62] Phase 1/2	*n* = 55 Cohort A R/R, *N* = 20 Cohort B (TN, ASCT not eligible) *n* = 24, Cohort C TN, ASCT not eligible) *N* = 12	Acalabrutinib + venetoclax + obinutuzumab	Cohort A: 24 m Cohort B: 20 m Cohort C: 9 m	Cohort A: ORR 86%, CR 75%, 2 yrs PFS75%, OS 86% Cohort B: ORR 88% CR 83%, 2 yrs PFS 78%, OS 96%, Cohort C: ORR 100%, CR 100%, 1 yr PFS 100%, OS100%	Most common all Gr AEs: bruising 41%; diarrhea 29%; nausea 25%.	AVO is a well-tolerated and effective regimen in pts with R/R and TN MCL, with high rates of MRD-CR in TN MCL.
Ruan et al. 2026 [63] Phase 2	*n* = 34 TN MCL	ALR (*N* = 24) or ALO (*N* = 10)	ALR: 53 m ALO: 25 m	ALR: ORR 100%, CR 83%. 3 yrs, PFS 76%, OS 91% ALO: ORR 90%, CR 90%, 2 yrs PFS 100%, OS 100%	Gr ¾ toxicities: ALR—asymptomatic neutropenia 33%, anemia 4%, thrombocytopenia 4%. ALO—asymptomatic neutropenia 40%, anemia 0%, thrombocytopenia 30%.	ALR and ALO are safe and active regimens feasible as a time-limited initial therapy for patients with MCL.

Abbreviations: AEs adverse events, AF—atrial fibrillation, ALO—acalabrutinib + lenalidomide + obinutuzumab, ALR—acalabrutinib + lenalidomide + rituximab, ART—acalabrutinib + venetoclax + rituximab, AVO—acalabrutinib + venetoclax + obinutuzumab, ASCT—autologous stem cell transplantation, BID—twice daily, BR—bendamustine plus rituximab, BTKi—Bruton’s tyrosine kinase inhibitor, CR—complete response, m—months, Gr—grade, MCL—mantle cell lymphoma, MRD—measurable residual disease, ORR—overall response rate, OS—overall survival, PFS—progression-free survival, Pts—patients, R/R—relapsed/refractory, TN—treatment-naïve.

## Data Availability

No new data were created or analyzed in this study. Data sharing is not applicable to this article.
